# Two‐Stage Sequential Fermentation of Oat Drink Using *Saccharomyces boulardii* and *Lactiplantibacillus plantarum*: A Promising Strategy for Developing Paraprobiotic‐Probiotic Food

**DOI:** 10.1002/fsn3.72268

**Published:** 2026-08-24

**Authors:** Nima Babolanimogadam, Hassan Gandomi, Afshin Akhondzadeh Basti, Mohammad J. Taherzadeh, Sara Ahadzadeh, Seyed Hasan Sajjadi Alhashem, Melika Farzaneh

**Affiliations:** ^1^ Department of Food Hygiene and Quality Control, Faculty of Veterinary Medicine University of Tehran Tehran Iran; ^2^ Swedish Centre for Resource Recovery University of Borås Borås Sweden; ^3^ Department of Pharmacology and Toxicology, School of Pharmacy Ardabil University of Medical Sciences Ardabil Iran; ^4^ Department of Food Science and Technology Islamic Azad University, Science and Research Branch Tehran Iran; ^5^ Department of Food Science and Technology, Shahr‐e‐Qods Branch Islamic Azad University Tehran Iran

**Keywords:** functional food, oat drink, paraprobiotic, plant‐based food, probiotic, sequential fermentation

## Abstract

Developing probiotic plant‐based products with high probiotic count, functionality, and sensory appeal remains challenging. In this study, oat drinks pretreated with NaOH, phosphoric acid, α‐amylase, sprouting, or their combinations were fermented using *Saccharomyces boulardii* alone, *Lactiplantibacillus plantarum* 299v alone, or a sequential process in which the live cells of *S. boulardii*‐fermented oat drink were heat‐inactivated (paraprobiotic) and subsequently fermented with *L. plantarum* . The results showed that the α‐amylase pretreatment yielded the highest *S. boulardii* growth (7.04 Log CFU mL^−1^; *p* < 0.05). Moreover, in both single‐strain and sequential fermentations, oat drinks pretreated with alkaline and alkaline‐α‐amylase combinations showed significantly lower *L. plantarum* counts than the other treatments (*p* < 0.05). Sequential fermentation of sprouted‐α‐amylase treated oat drink yielded the highest phenolic content (381.67 mg GAE L^−1^; *p* < 0.05). Both the oat drink fermented with *L. plantarum* alone and that produced by sequential fermentation achieved high probiotic counts (> 10 Log CFU mL^−1^) and favorable sensory scores (> 7), but sequentially fermented oat drink outperformed all other evaluated treatments, exhibiting balanced acidity, superior antioxidant activity, and negligible alcohol (< 0.5%). To our knowledge, this is the first report integrating paraprobiotic‐probiotic concepts into oat drink, offering a novel, non‐alcoholic strategy to enhance functionality, consumer appeal, and health‐promoting qualities in plant‐based foods.

## Graphical abstract

This study presents a novel two‐stage sequential fermentation for developing a functional oat drink with dual paraprobiotic‐probiotic properties. Oat substrates pretreated with acid, alkaline, enzyme, sprouting, or their combinations were first fermented with *S. boulardii*, heat‐inactivated, and then re‐fermented with live *L. plantarum* 299v. The sequential process delivered high probiotic counts, enhanced phenolic content and antioxidant activity, negligible ethanol, and favorable sensory scores, outperforming conventional single‐strain fermentations.

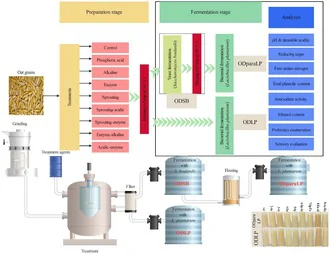

## 1 Introduction

Nowadays, the production and consumption of functional foods have witnessed a notable increase due to their health‐promoting properties and nutritional functions (Corbo et al. 2014). Probiotic‐containing foods represent a key category of functional foods (Ferreira et al. 2023), offering diverse benefits to consumers. These advantages encompass the production of microbial metabolites and favorable compounds that facilitate digestion and/or modify macromolecules and anti‐nutritional substances in food (Marco et al. 2017; Yang et al. 2023; Yu et al. 2023). Furthermore, when administered in appropriate quantities in their living forms, these microorganisms can positively impact host health (Marco et al. 2017). Additionally, fermentation plays a pivotal role in enhancing the nutritional value, sensory properties, and shelf life of food products (Wronkowska et al. 2022).

Plant‐based drinks or beverages, water‐soluble extracts from a variety of plants including cereals, pseudocereals, legumes, and nuts, are produced through diverse treatments and are regarded as healthful and eco‐friendly alternatives (Łopusiewicz 2023; Rasika et al. 2021). Their rising popularity can be attributed to their functional constituents such as antioxidant compounds, dietary fiber, prebiotics, minerals, and vitamins (Sethi et al. 2016), which depend on the plant source and type of pre‐treatments (Babolanimogadam et al. 2023), and play an important role in health promotion (Ferreira et al. 2023). Furthermore, plant‐based drinks are free of components that are associated with dairy‐related health issues, such as cow's milk proteins, lactose, allergens, and cholesterol (Silva et al. 2020). In addition, replacing foods rich in saturated fat with healthier unsaturated fat sources is consistent with the World Health Organization (WHO) recommendations for reducing the risk of cardiovascular disease (World Health Organization 2023).

Oat drink, a cereal‐based drink, is recognized as an excellent source of nutrients and health‐promoting components, including unsaturated fatty acids, high‐quality proteins and amino acids, vitamins, antioxidants (e.g., tocopherols, phenolic compounds, and avenanthramides), dietary fibers (e.g., β‐glucan), and minerals (Łopusiewicz 2023; Silva et al. 2020). Furthermore, prior research has indicated that fermentation enhances the functional attributes of oat drink, including an increase in the bioavailability of phenolic compounds, an improvement in antioxidant activity, and the production of bioactive peptides and vitamins, with the extent of these enhancements depending on the microbial strains employed, as well as fermentation time and temperature (Dhakal et al. 2024; Djorgbenoo et al. 2023). Conversely, oat drink constituents may facilitate microbial proliferation during fermentation (Gupta and Bajaj 2017). It is documented that the innovation of fermented oat drink commenced three decades ago, predominantly employing lactic acid bacteria; however, the utilization of other microorganisms, such as *Saccharomyces boulardii*, has been explored (Bocchi et al. 2021; Yang et al. 2023).

The probiotic *Lactiplantibacillus plantarum* 299v ( *L. plantarum* 299v), a Gram‐positive, microaerophilic/facultative bacterium isolated from human intestinal mucosa, is regarded as the most extensively documented *L. plantarum* strain globally (Nordström et al. 2021). It is also a principal strain for fermenting plant‐based foods (Vinderola et al. 2017), and different studies have utilized it to ferment oat and other plant‐based products, highlighting its efficacy in probiotic food production and health‐promoting properties (Skrzypczak et al. 2024; Ziarno et al. 2025).

*Saccharomyces boulardii* CNCM I‐745 (*S. boulardii*), as a probiotic yeast, has been applied in fermentation processes to enrich flavor and nutrition, augment vitamin B complex, and enhance mineral bioavailability by reducing phytic acid levels in functional probiotic foods such as rice bran, vegetable juice, and non‐alcoholic beer (Değirmencioğlu et al. 2016; Rekha and Vijayalakshmi 2010; Ryan et al. 2011; Senkarcinova et al. 2019). Additionally, studies suggest that *S. boulardii*, along with other yeasts, can support probiotic bacterial growth during the co‐fermentation of plant‐based foods by secreting enzymes or metabolizing different compounds such as polysaccharides, proteins, and lipids (Dong et al. 2026; González‐Figueredo et al. 2021; Polat Kaya et al. 2025; Wang et al. 2022).

Although the functional and health‐promoting attributes of live probiotics have been extensively documented, inactivated forms of these microorganisms, referred to as “paraprobiotics” or “ghost probiotics,” have likewise been shown to confer host benefits. The underlying mechanisms are multifaceted and include immunomodulation, competitive exclusion of pathogens through enhanced adhesion to the intestinal epithelium, and the release of bioactive metabolites (De Almada et al. 2016). In a more targeted application, heat‐killed cells of *S. boulardii* were demonstrated to preserve intestinal integrity, modulate the immune response, and prevent bacterial translocation as well as intestinal lesions in a murine model, thereby validating the therapeutic potential of this specific paraprobiotic (Generoso et al. 2011). In contrast to their live counterparts, paraprobiotics are particularly suited for functional foods that pose technological challenges, offering advantages in food processing, thermal stability, storage, transport, and extended shelf life (De Almada et al. 2016).

Previous studies on plant‐based fermentation have primarily focused on the use of either lactic acid bacteria or yeasts as individual starter cultures, while a limited number have examined their co‐fermentation using live microorganisms. However, to the best of our knowledge, no study has yet reported a sequential fermentation strategy for a food matrix in which a yeast is first cultivated, subsequently heat‐inactivated to produce a paraprobiotic biomass, and then used as the basis for fermentation with a live probiotic bacterium. The present study, part of a broader project on developing paraprobiotic‐probiotic oat drink, focuses on sequential fermentation. The oat drinks, which were prepared with various pretreatments, including phosphoric acid, alkaline, α‐amylase, sprouting, and their combinations, were first fermented with S. boulardii. After thermal inactivation, the paraprobiotic‐enriched substrate was subsequently fermented with *L. plantarum* 299v. The aim of this investigation was the evaluation of effects of different pretreatment on the nutritional, functional, and sensory properties of oat drinks produced by single‐strain and two‐stage sequential fermentation, and to compare these two fermentation strategies with one another.

## 2 Materials and Methods

### 2.1 Materials

Lyophilized capsules of *Saccharomyces cerevisiae* var. *boulardii* CNCM I‐745 (*S. boulardii*; Yomogi, Mutaflor Co., Australia) and *Lactiplantibacillus plantarum* 299v ( *L. plantarum* ; DSM 9843; *Probi Mage*, Sweden) were purchased from a local drugstore in Lund, Sweden. All other chemicals were purchased from Merck (Darmstadt, Germany).

### 2.2 Preparation and Enumeration of Probiotics

Capsules containing dried *L. plantarum* and *S. boulardii* cells were rehydrated and cultured in 10 mL of de Man, Rogosa, and Sharpe (MRS) broth and yeast peptone dextrose (YPD) broth media, respectively, and incubated at 37°C and 30°C for 24 h, respectively (Călinoiu et al. 2019). To ensure full metabolic activity and viability, the microorganisms were activated through three successive subcultures in their respective media. Following activation, microbial suspensions were standardized to an optical density of 1 at 600 nm (OD_600_ = 1) using a UV–Vis spectrophotometer (DR‐5000 model, manufactured by HACH, Germany) and phosphate‐buffered saline (PBS, 0.05 M, pH 7.0) to ensure consistent inoculum density across experiments. *L. plantarum* and *S. boulardii* were enumerated using the plate count method on MRS and YPD agar, respectively. Plates were incubated for 48 h at 37°C for *L. plantarum* and 30°C for *S. boulardii*. The results were expressed as log_10_ CFU per mL (Log CFU mL^−1^).

### 2.3 Formulation of Oat Drink

Oat drink preparation by using whole‐oat grains followed methods reported in our previous study (Babolanimogadam et al. 2023). Briefly, different formulations included phosphoric acid‐treated, alkaline‐treated, and enzyme‐treated oat drink, produced by mixing the milled oat (smaller than 0.5 mm) with distilled water (1:9 w/v) containing phosphoric acid (5 mL; 85%), NaOH (5 mL; 40% w/v), or α‐amylase (200 mg; activity 11,500 U/g, from *Aspergillus oryzae*, DSM Nutritional Products, Heerlen, Netherlands), respectively. In addition, a control and a sprouted treatment were prepared using milled oat (smaller than 0.5 mm) or milled pre‐sprouted oat grain (for sprouted treatment), each mixed with distilled water at a ratio of 1:9 (w/v). Sprouting was performed by soaking oat grains in distilled water (1:6 w/v) at 25°C for 48 h, followed by germination in a dark chamber at 18°C with 95% relative humidity for 96 h, with periodic water spraying to maintain moisture. After germination, the sprouted grains were dried at 50°C for 24 h and milled. Additionally, different combinations of treatments, including sprouting–acidic, sprouting–enzyme, enzyme–alkaline, and acidic–enzyme, were prepared. Slurries were manually filtered, adjusted to pH 6.7, divided into separate bottles (two groups per treatment), heated at 90°C for 10 min and stored at 4°C for subsequent analysis. To verify the sterility of the base oat drinks prior to fermentation, aliquots of the heat‐treated samples were plated on Plate Count Agar (PCA), MRS agar, and YPD agar, and incubated under both aerobic and anaerobic conditions at 30°C and 37°C for 72 h. The absence of any visible colonies confirmed the adequacy of the heat treatment in eliminating the natural background microflora, ensuring that subsequent fermentations were initiated exclusively by the inoculated starter cultures.

### 2.4 Fermentation of Oat Drink

**FIGURE 1:**
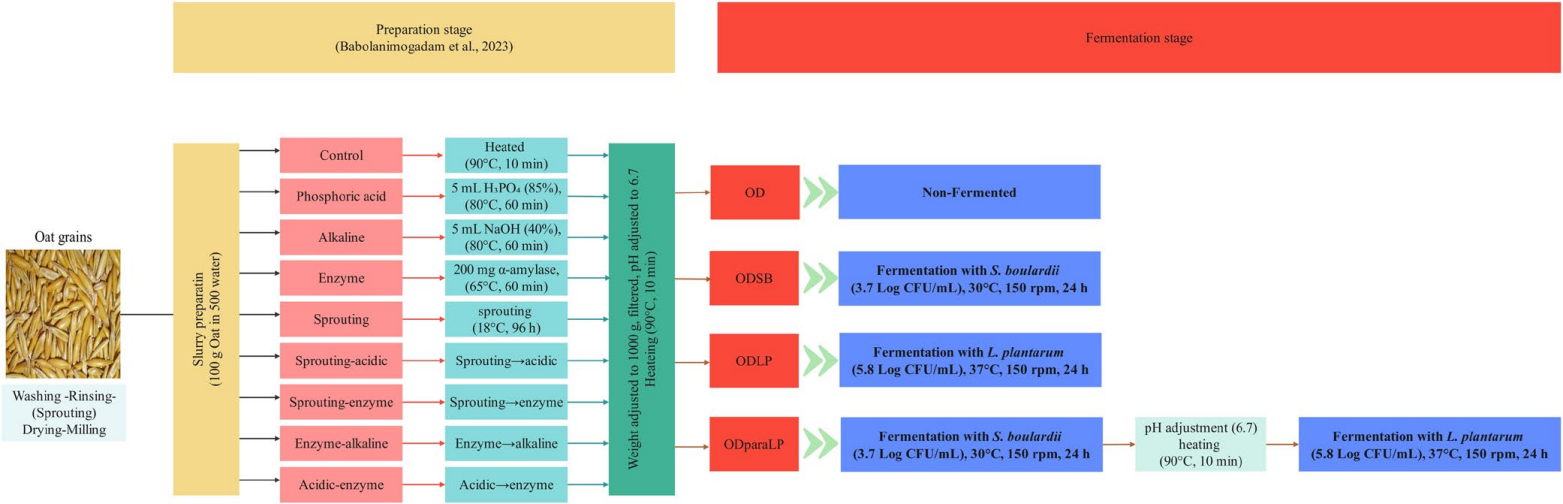
The schematic diagram of preparation of different oat drink treatments (OD: Non‐fermented oat drink, ODSB: Oat drink fermented with *S. boulardii*, ODLP: Oat drink fermented with *L. plantarum* , and ODparaLP: Sequential fermented oat drink).

A schematic diagram of the oat drink preparation for different treatments is presented in Figure 1. Treatments included non‐fermented oat drink (*n* = 9), oat drink fermented with *L. plantarum* (*n* = 9), oat drink fermented with *S. boulardii* (*n* = 9), and sequential fermented oat drink (*n* = 9). The oat drink fermented with *L. plantarum* group was inoculated with *L. plantarum* at an initial concentration of 5.8 Log CFU mL^−1^, and the oat drink fermented with *S. boulardii* with *S. boulardii* at an initial concentration of 3.7 Log CFU mL^−1^. These were incubated at 37°C and 30°C, respectively, with shaking at 150 rpm for 24 h. The shaking was applied solely to prevent sedimentation of the oat drink solids and maintain sample uniformity. For the sequentially fermented oat drink, after fermentation of oat drink with *S. boulardii*, the pH was adjusted to 6.7 and heated at 90°C for 10 min. The efficacy of this heat inactivation step was rigorously confirmed by plating the heat‐treated samples on YPD agar and incubating at 30°C for 48 h, which resulted in no colony formation. This confirmed the complete absence of viable *S. boulardii* cells before the subsequent inoculation with *L. plantarum* . Finally, prepared paraprobiotic oat drinks were inoculated with *L. plantarum* (5.8 Log CFU mL^−1^) and incubated at 37°C for 24 h.

### 2.5 Measurement of pH and Acidity

The pH of the oat drink treatments was measured using a pH meter. Titratable acidity was determined by titrating 10 mL of sample against 0.1 N NaOH, with continuous swirling, until the pH reached 8.2. The titratable acidity was calculated using the following equation: 1.0 mL of 0.1 N NaOH = 0.0090 g of lactic acid (Gupta and Bajaj 2017). The data were expressed as g lactic acid/100 mL of the sample.

### 2.6 Reducing Sugar

The reducing sugar content of the samples was measured by the 3,5‐Dinitrosalicylic acid (DNS) method (Jain et al. 2020). Briefly, 1 mL of diluted samples was mixed with 1 mL of freshly prepared DNS reagent, incubated in a water bath for 15 min at boiling temperature, cooled to 25°C, and finally adjusted to 10 mL using distilled water. DNS reagent solution was prepared by mixing 1.5 g of DNS in 30 mL of NaOH solution (2 M) and then mixing with sodium potassium tartrate solution (45 g in 75 mL of distilled water) and finally adjusting the volume to 150 mL with distilled water. The optical density of the samples was measured at 540 nm. The calibration curve was obtained using glucose (0.0025%–0.1%), and the results were expressed as percent.

### 2.7 Free Amino Nitrogen (FAN)

FAN was determined according to the ninhydrin method described by Abernathy et al. (2009), with some modifications. Ten mL of samples were mixed with 100 mL of distilled water, stored at 4°C for 4 h, and centrifuged at 6000 × *g* for 5 min at 25°C. Aliquots of 2 mL of supernatant were mixed with 1 mL ninhydrin reagent. The ninhydrin reagent was prepared by mixing 4.97 g of disodium hydrogen phosphate dihydrate, 6 g of potassium dihydrogen phosphate, 0.3 g of fructose, and 0.5 g of ninhydrin and making up the volume to 100 mL with distilled water. The mixture was boiled for 15 min, cooled in an ice bath for 20 min, mixed with 5 mL of dilution reagent, and incubated at 25°C for 20 min. Dilution reagent was prepared by dissolving 2 g of potassium iodate in 600 mL of distilled water and 400 mL of 96% ethanol. Absorbance was measured at 570 nm. Standard glycine (107.2 mg/100 mL) was used as a reference. The results were expressed as mg FAN per L of sample (mg FAN L^−1^).

### 2.8 Total Phenolic Content (TPC) and Antioxidant Activity (AOA)

The extraction of bioactive compounds was done according to the previous study (Aparicio‐García et al. 2021; Babolanimogadam et al. 2023). For this purpose, 1 mL of oat drink samples was mixed with acidified methanol (methanol:water:HCl 80:19.9:0.1 v/v), homogenized at 12000 rpm for 2 min, and agitated for 16 h at 25°C. Following centrifugation at 6000 × *g* for 15 min at 4°C, the collected supernatants were used for the determination of TPC and AOA. The TPC was determined using the Folin–Ciocalteu's reagent method (Best et al. 2020). A 100 μL aliquot of oat drink extracts was mixed with 750 μL of Folin–Ciocalteau reagent (0.2 N) and incubated for 5 min at 25°C. Then, 750 μL of sodium carbonate (7.5%) was added and incubated in a water bath at 40°C for 30 min. The absorbance was recorded at 725 nm, and the results were expressed as mg gallic acid equivalents per liter of the sample (mg GAE L^−1^) by plotting the standard curve using different concentrations of gallic acid as standard. For the evaluation of antioxidant activity, 50 μL of extracts were mixed with 950 μL of 1,1‐diphenyl‐2‐picrylhydrazyl (DPPH) solution (65 μmol L^−1^ in methanol) and incubated at 25°C for 10 min. The absorbance was recorded at 515 nm. DPPH solution without the sample was used as a blank (Rahmani et al. 2021). Butylated hydroxytoluene (BHT) was used as a standard antioxidant. The results were expressed as mg BHT eq L^−1^ of the samples.

### 2.9 Ethanol Production

The ethanol content of the fermented oat drink was analyzed using high‐performance liquid chromatography (HPLC). A 20 μL aliquot of diluted samples, filtered through a 0.22 μm membrane filter, was injected into an HPX–87H column (Bio–Rad, Hercules, CA, USA). A 5 mM sulfuric acid solution was used as the mobile phase. The column temperature was maintained at 30°C with a 0.4 mL/min flow rate. The ethanol concentration was determined at 214 nm. A calibration curve of pure ethanol was obtained prior to the experimental work (*R* ^2^ > 0.996).

### 2.10 Sensory Evaluation

The sensory evaluation of oat drink was performed by a group of 30 trained panelists, including students and staff of Ardabil University of Medical Sciences (aged between 19 and 40 years). The sensory attributes of color, aroma, flavor, and overall acceptability of samples (pH adjusted to 6.7) were scaled using a 9‐point hedonic scale (1 = dislike extremely, 2 = dislike very much, 3 = dislike moderately, 4 = dislike slightly, 5 = neither like nor dislike, 6 = like slightly, 7 = like moderately, 8 = like very much, and 9 = like extremely). The samples were served in clear plastic cups containing 25 mL of product with a random three‐digit code at 25°C under normal daylight conditions. Filtered water was provided for panelists to rinse their mouths to minimize residual effects. Informed consent was obtained from all panelists. This research study involving human participants was in accordance with the ethical standards of the institutional and national research committee and with the 1964 Helsinki Declaration and its later amendments or comparable ethical standards. The University/regional research ethics committee of the Ardabil University of Medical Sciences approved this study (IR.ARUMS.REC.1399.287).

### 2.11 Statistical Analysis

All analyses of fermented oat drink treatments were performed in triplicate, and the results were expressed as means ± standard deviations. Data were statistically analyzed by one‐way analysis of variance (ANOVA) using SPSS for Windows (Version 22.0, SPSS Inc., Chicago, IL, USA). Duncan's test was applied to determine significant differences (*p* < 0.05). The sensory evaluation results were analyzed by the nonparametric Kruskal–Wallis test, followed by paired comparison using the Mann–Whitney *U* test.

## 3 Results and Discussion

### 3.1 pH Value and Titratable Acidity

**FIGURE 2:**
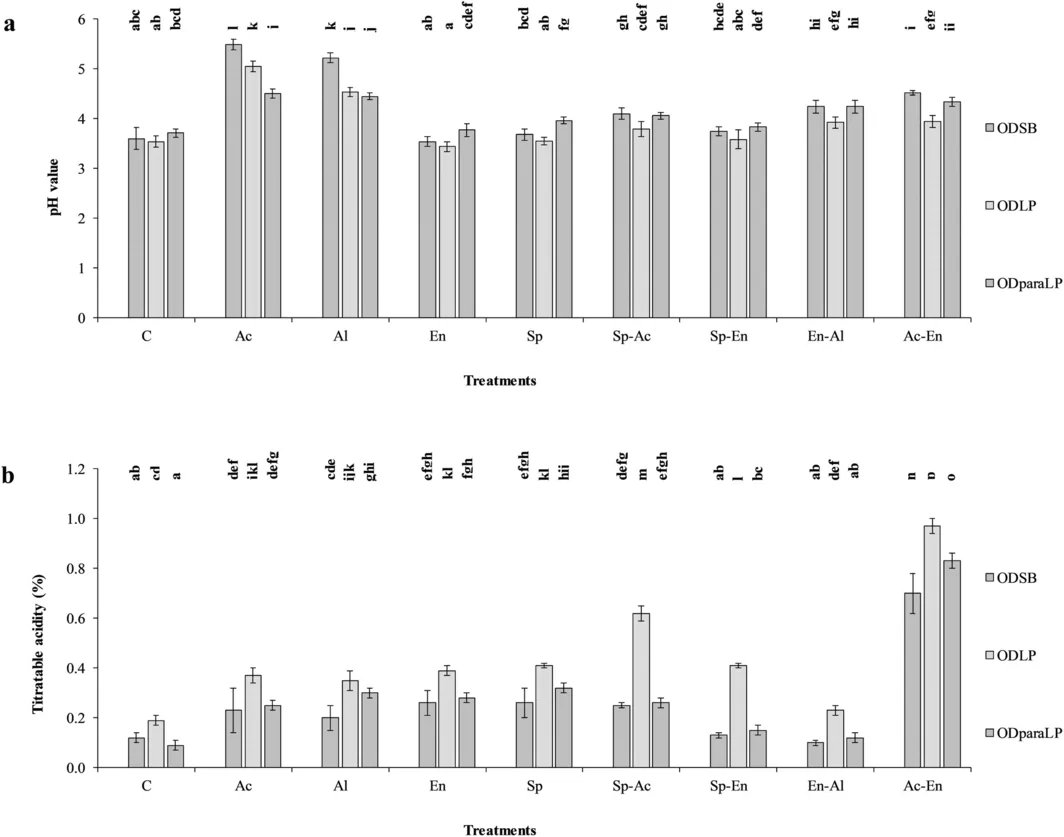
The pH value (a) and titratable acidity (b) of oat drink fermented with *S. boulardii* (ODSB), oat drink fermented with *L. plantarum* (ODLP), and sequential fermented oat drink with *S. boulardii* and *L. plantarum* (ODparaLP); Treatments: Control (C), phosphoric acid (Ac), alkaline (Al), α‐amylase enzyme (En), sprouting (Sp), sprouting‐phosphoric acid (Sp‐Ac), sprouting‐α‐amylase enzyme (Sp‐En), α‐amylase enzyme‐alkaline (En‐Al), and phosphoric acid‐α‐amylase enzyme (Ac‐En). Different letters on the graph indicate significant differences at the 5% level.

Probiotics produce lactic acid, which not only alters the pH value of the media and affects the viability of microorganisms but also influences the acceptability of the product by consumers due to its impact on the functional and organoleptic characteristics of food (Gupta and Bajaj 2017). The pH value and titratable acidity of oat drink after fermentation are shown in Figure 2a,b, respectively. The pH values of all treatments exhibited decreasing trends, while their acidity increased during the 24 h fermentation period. The pH value and titratable acidity of oat drink fermented with *S. boulardii* ranged from 3.54 (enzyme treatment) to 5.49 (acidic treatment), and from 0.1% (enzyme–alkaline treatment) to 0.7% (acidic–enzyme treatment), respectively. Similarly, the pH values for oat drink fermented with *L. plantarum* and sequentially fermented oat drink ranged from 3.44 (enzyme treatment) to 5.05 (acidic treatment), and titratable acidity ranged from 0.09% (control; sequentially fermented oat drink) to 0.97% (acidic–enzyme; oat drink fermented with *L. plantarum* ), respectively. These results are consistent with previous studies (Bernat et al. 2015; Salmerón et al. 2015), which found that the pH value of fermented oat drink with probiotic lactic acid bacteria decreased, while acidity increased during fermentation. However, Rathore et al. (2012) reported higher acidity in cereal beverages compared to the results of this study. These differences may be attributed to the cereal‐to‐water ratio, the type and inoculation size of probiotics, fermentation time and temperature, and the type of pretreatments. Additionally, it was reported that a higher initial concentration of the reducing sugar could result in higher lactic acid production (Salmerón et al. 2015), as corroborated by Gupta and Bajaj (2017), who reported a relatively high lactic acid content (0.85%) after 48 h of fermentation of oat slurries containing 3% honey.

The pH value of oat drink fermented with *S. boulardii* ranged from 3.54 (enzyme treatment) to 5.49 (acidic treatment), and the pH value of sequentially fermented oat drink treatments ranged from 3.71 (control treatment) to 4.5 (acidic treatment), which was significantly (*p* < 0.05) higher than that of oat drink fermented with *L. plantarum* treatments. Meanwhile, the acidity of all oat drink fermented with *L. plantarum* treatments (except for alkaline treatment) was significantly higher than that of sequentially fermented oat drink and oat drink fermented with *S. boulardii* treatments (*p* < 0.05). These results not only demonstrate the higher lactic acid production capability of *L. plantarum* compared to *S. boulardii*, but also show that pre‐fermentation of oat drink with yeast resulted in a higher pH and lower lactic acid production. Similarly, higher lactic acid production by probiotic bacteria after 24 h of co‐fermentation of soy drink compared to *S. boulardii* was previously reported by Rekha and Vijayalakshmi (2010). Additionally, Chan et al. (2021) reported lower acid production during the 24 h co‐fermentation of a coffee beverage by *S. boulardii* and *L. rhamnosus* GG, due to the citric acid consumption by the yeast, which limited bacterial growth. The consumption of reducing sugar, L‐glutamic acid, and L‐alanine as precursors of lactic acid by the yeast (Chan et al. 2021) could reduce post‐acidification by probiotic bacteria. Furthermore, using dry yeast cells as a nitrogen source induced lower lactic acid production and productivity (Ooi and Wu 2015), possibly due to the limited release of nitrogen compounds from dry yeast cells and the interference of trehalose, the carbohydrate storage form of yeast, with glucose digestion, thereby negatively affecting lactic acid production. The higher pH value, despite the higher acidity of treatments prepared using phosphoric acid, can be attributed to the buffering properties induced by the produced phosphoric acid salts (Mennah‐Govela and Bornhorst 2021), which support the growth of probiotics, unlike treatments prepared in alkaline conditions (alkaline and enzyme–alkaline treatments).

### 3.2 Reducing Sugar and FAN Content of Oat Drink

**FIGURE 3:**
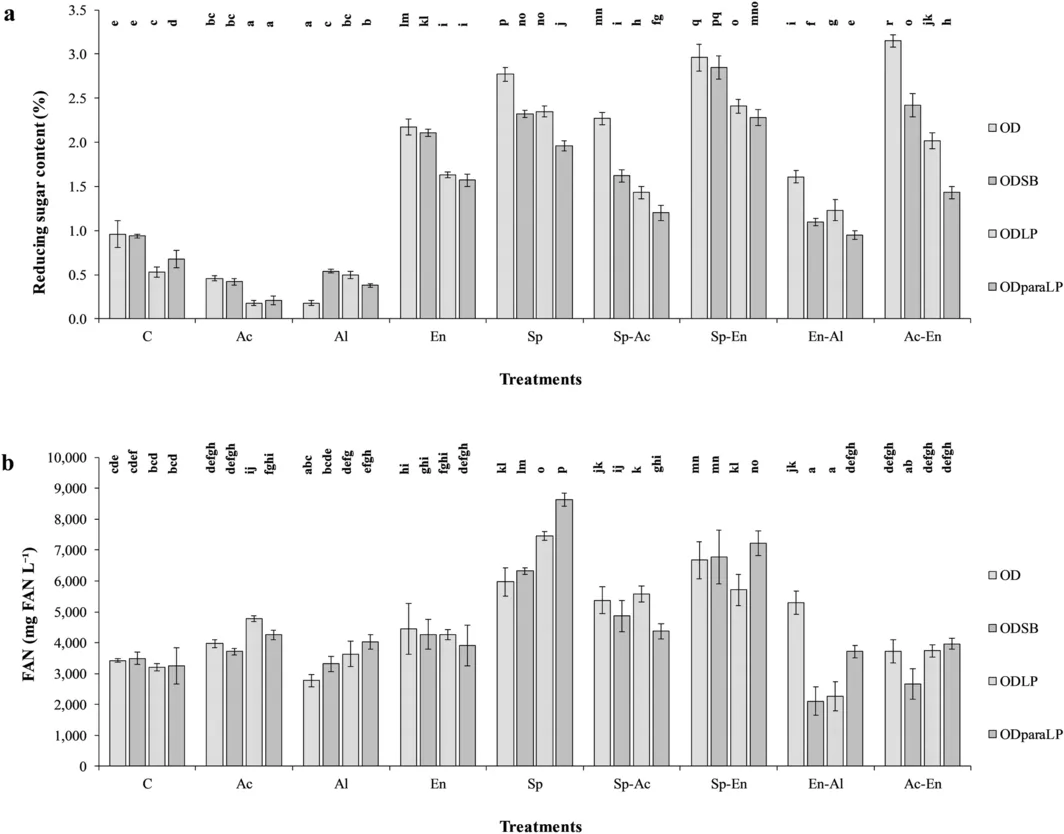
The reducing sugar (a) and free amino nitrogen (b) of non‐fermented oat drink (OD), oat drink fermented with *S. boulardii* (ODSB), oat drink fermented with *L. plantarum* (ODLP), and sequential fermented oat drink with *S. boulardii* and *L. plantarum* (ODparaLP); Treatments: Control (C), phosphoric acid (Ac), alkaline (Al), α‐amylase enzyme (En), sprouting (Sp), sprouting‐phosphoric acid (Sp‐Ac), sprouting‐α‐amylase enzyme (Sp‐En), α‐amylase enzyme‐alkaline (En‐Al), and phosphoric acid‐α‐amylase enzyme (Ac‐En). Different letters on the graph indicate significant differences at the 5% level.

The reducing sugar and FAN content of fermented oat drink are shown in Figure 3a,b, respectively. The results indicated that the reducing sugar content of oat drink fermented with *L. plantarum* and sequentially fermented oat drink was significantly (*p* < 0.05) decreased during fermentation compared to non‐fermented oat drink treatments, except for the alkaline treatments. Also, the reducing sugar content of most two‐stage prepared oat drink treatments (sprouting–acidic, enzyme–alkaline, and acidic–enzyme treatments), along with sprouted treatment, was significantly (*p* < 0.05) decreased during oat drink fermentations with *S. boulardii*. These results indicated that the reducing sugar was consumed by probiotic yeast and bacteria after 24 h of fermentation. Additionally, the reducing sugar content of most sequentially fermented oat drink treatments was significantly (*p* < 0.05) lower than that of oat drink fermented with *S. boulardii* and oat drink fermented with *L. plantarum* due to the two‐step fermentation and the growth‐supporting properties of yeast. Previous studies have reported varying rates of the reducing sugar consumption by probiotics after 24 h of fermentation (Kedia et al. 2007; Rozada‐Sánchez et al. 2008), consistent with the present findings. The results also showed an increase in the reducing sugar after *S. boulardii* fermentation, aligning with the reports of González‐Figueredo et al. (2021). This increase may be related to the starch or other carbohydrate digestion abilities of the yeast. However, previous studies did not report a similar ability for *L. plantarum* . Interestingly, the unexpected increase in the reducing sugar content observed in the alkaline treatment of oat drink fermented with *L. plantarum* , compared to non‐fermented oat drink, may be attributed to the cleavage of sugars and other macromolecules during fermentation or the action of glycoside hydrolase produced by *L. plantarum* . The results also demonstrated that the higher initial reducing sugar content of acidic–enzyme and sprouting–enzyme treatments enhanced the reducing sugar consumption by *L. plantarum* .

FAN consists of amino acids and small peptides, which are produced by the action of proteinase and peptidase enzymes of microorganisms and is also generated during germination (Jiang et al. 2023). Plant‐based nitrogen, which is assimilable by yeast, mainly comprises amino acids, ammonium ions, and (di and tri) peptides (Jiang et al. 2023), which are mostly formed during oat drink pretreatments. The FAN content of non‐fermented oat drink ranged from 2774 mg FAN L^−1^ (alkaline treatment) to 6674 mg FAN L^−1^ (sprouting–enzyme treatment). The results indicated that the FAN content of the sprouting treatment was significantly (*p* < 0.05) higher than other treatments, which is in agreement with the findings of Tian et al. (2010), who reported an increase in FAN content during germination. Moreover, the higher FAN content of sprouting–enzyme treatment of non‐fermented oat drink may be related to higher yield of oat drink leading to higher starch degradation. During *S. boulardii* fermentation, the FAN content of enzyme–alkaline and acidic–enzyme treatments significantly decreased, while other treatments showed no significant change (*p* < 0.05). Additionally, oat drink fermented with *L. plantarum* and sequentially fermented oat drink treatments exhibited varying increasing and decreasing FAN trends during *L. plantarum* fermentation. The sprouted treatment showed the highest FAN content after bacterial fermentation (oat drink fermented with *L. plantarum* and sequentially fermented oat drink).

Similar FAN fluctuations have been reported by different studies (Gharbi Yahyaoui et al. 2017; Salmerón et al. 2015; Zhang et al. 2015), attributed to protein degradation by endo/exoproteases and amino‐acid release during cell lysis. We found no previous reports on exoprotease activity of *L. plantarum* 299v. The observed FAN increase after 24 h of *L. plantarum* fermentation may reflect endoproteinase activity and cell‐lysis release, but further study is needed.

### 3.3 TPC and AOA of Oat Drink

**FIGURE 4:**
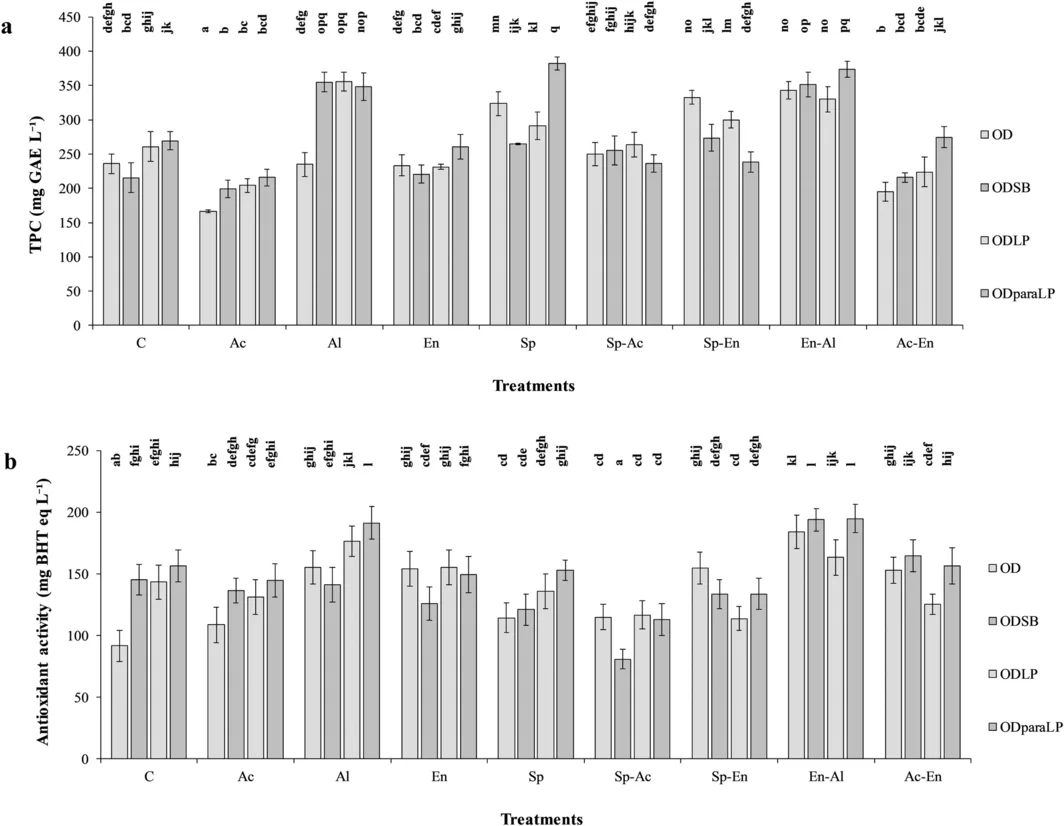
The total phenolic content (a) and antioxidant activity (b) of non‐fermented oat drink (OD), oat drink fermented with *S. boulardii* (ODSB), oat drink fermented with *L. plantarum* (ODLP), and sequential fermented oat drink with *S. boulardii* and *L. plantarum* (ODparaLP); Treatments: Control (C), phosphoric acid (Ac), alkaline (Al), α‐amylase enzyme (En), sprouting (Sp), sprouting‐phosphoric acid (Sp‐Ac), sprouting‐α‐amylase enzyme (Sp‐En), α‐amylase enzyme‐alkaline (En‐Al), and phosphoric acid‐α‐amylase enzyme (Ac‐En). Different letters on the graph indicate significant differences at the 5% level.

Phenolic compounds, considered the main antioxidants in oat grains and products, are predominantly cell wall‐bound (Bei et al. 2017; Soycan et al. 2019). The extraction yield of these compounds in oat products depends on different treatments such as sprouting, enzymes, alkali or acid pretreatments, and fermentation (Babolanimogadam et al. 2023; Bei et al. 2018). The TPC and AOA of the various oat drink treatments are shown in Figure 4a,b, respectively. The TPC of fermented treatments ranged from 199.33 mg GAE L^−1^ (acidic treatment; oat drink fermented with *S. boulardii*) to 381.67 mg GAE L^−1^ (sprouted treatment; sequentially fermented oat drink). The results indicated that the fermentation methods had distinct effects on the TPC of oat drink produced by different treatments. Most treatments showed no significant change in TPC during yeast or bacterial fermentation; however, acidic and alkaline treatments showed an increase in TPC, whereas sprouting and sprouting–enzyme treatments decreased (oat drink fermented with *S. boulardii* and oat drink fermented with *L. plantarum* ). Moreover, sequentially fermented oat drink significantly enhanced TPC in all treatments except sprouting–enzyme treatment, which declined (*p* < 0.05). These findings are in agreement with the results of Bocchi et al. (2021), who reported that ferulic acid, unlike avenanthramides, increased in oat beverages after 48 h fermentation with *Lactobacillus* spp. and *Bifidobacterium* spp. due to the release of bounded phenolic compounds. Adebo and Medina‐Meza I (2020) review similar enhancements of oat phenolics by bacterial and yeast fermentation. The enhancing properties of phenolic compounds by *L. plantarum* 299v and *S. boulardii* during fermentation of plant‐based matrices have been shown by previous reports (Değirmencioğlu et al. 2016; Złotek et al. 2019). Variations across studies may reflect differences in pretreatment, microbial activity or species, fermentation time (Hur et al. 2014), and analytical or extraction methods (Cai et al. 2012). In the data obtained in this study, the higher TPC in some sequentially fermented oat drink treatments versus oat drink fermented with *L. plantarum* and oat drink fermented with *S. boulardii* correlated with their higher pH values, which may promote cell wall‐destroying enzyme activity by microorganisms (Hur et al. 2014), notwithstanding similar probiotic enumeration. Additionally, it may be due to the accumulation of phenolic compounds during the two‐stage fermentation of oat drink.

Antioxidants are molecules that relieve oxidative/nitrosative stress through reaction with free radicals (Kurutas 2015). The phenolic compounds and vitamin E are the main antioxidants in oats (Angelov et al. 2018). The AOA of fermented oat drink ranged from 81 mg BHT eq L^−1^ (sprouting–acidic; oat drink fermented with *S. boulardii*) to 194.67 mg BHT eq L^−1^ (enzyme–alkaline; sequentially fermented oat drink). Despite divergent TPC and AOA changes, the overall patterns (increasing, decreasing, and unchanged) were similar for both factors during the fermentation of oat drink with yeast or bacteria. For example, treatment C's AOA increased in oat drink fermented with *S. boulardii* and oat drink fermented with *L. plantarum* without significant TPC change; enzyme treatment saw decreased AOA during oat drink fermented with *S. boulardii* despite stable TPC; sprouted treatment showed decreased TPC in oat drink fermented with *L. plantarum* despite unchanged AOA. The phenomenon of inconsistency in the trends of TPC and AOA during oat drink fermentation was observed in other treatments as well.

Regardless of the effect of various pretreatments, the fermentations showed different effects on the AOA of the food matrix. Such TPC–AOA inconsistencies have been noted previously (Złotek et al. 2019). They found no AOA change despite increased TPC in legume sprouts fermentation with *L. plantarum* 299v. Moore et al. (2007) and Luana et al. (2014) also reported fermentation with *S. cerevisiae* and *L. plantarum* LP09, respectively, induced AOA changes in cereal matrices.

Previous studies have reported that an increase in TPC should be tantamount to an increase in AOA (Adebo and Medina‐Meza I 2020). However, due to the ambiguous relationship between the antioxidant activity and total phenolic compounds, as reviewed by a previous study (Đorđević et al. 2010), the AOA could change independently.

### 3.4 Probiotics Enumeration

**FIGURE 5:**
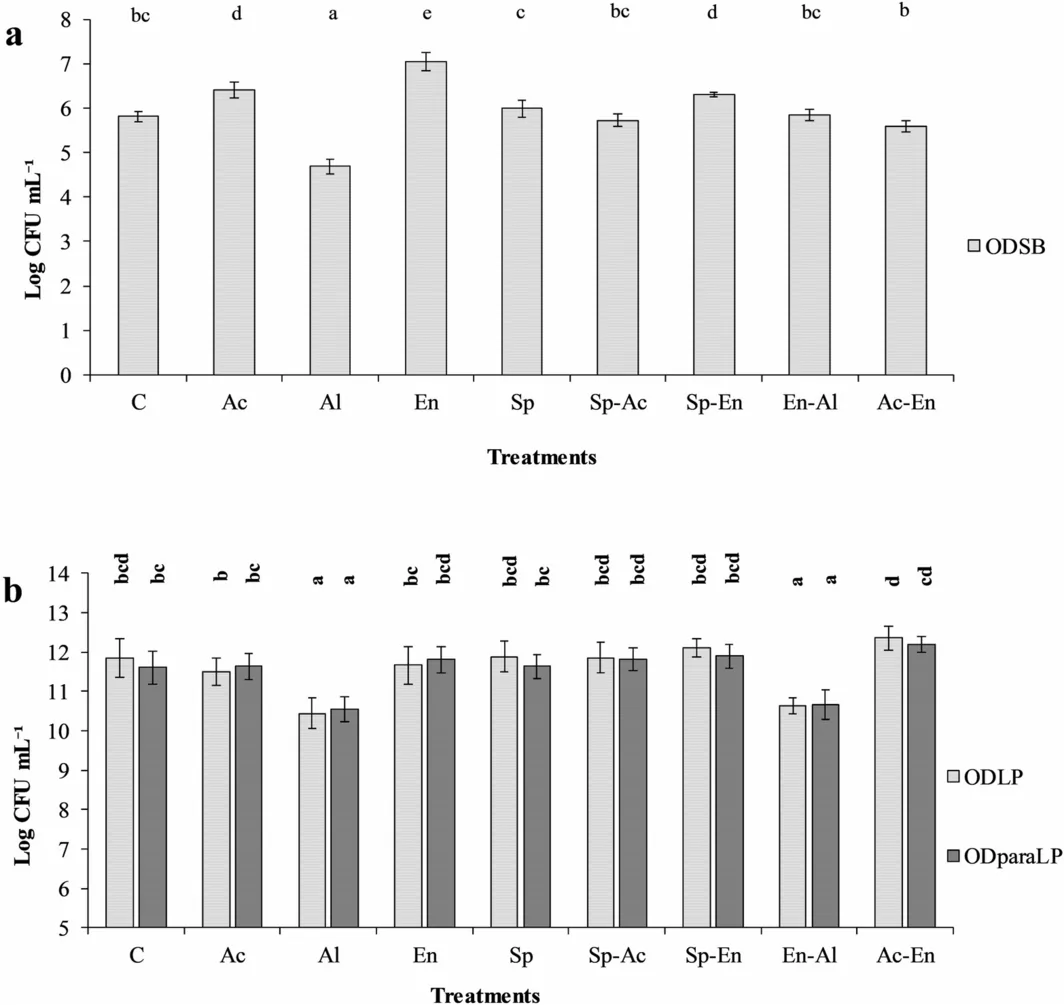
Enumeration of *S. boulardii* (a) and *L. plantarum* (b) during fermentation of oat drink with *S. boulardii* (ODSB), *L. plantarum* (ODLP), and successive fermentation with *S. boulardii* and *L. plantarum* (ODparaLP); Treatments: Control (C), phosphoric acid (Ac), alkaline (Al), α‐amylase enzyme (En), sprouting (Sp), sprouting‐phosphoric acid (Sp‐Ac), sprouting‐α‐amylase enzyme (Sp‐En), α‐amylase enzyme‐alkaline (En‐Al), and phosphoric acid‐α‐amylase enzyme (Ac‐En). Different letters on the graph indicate significant differences at the 5% level.

Some bioactive compounds are essential for developing new functional foods. In this study, phenolic compounds, the enumeration of viable probiotics (Gupta et al. 2010), and non‐viable probiotic cells could be considered as bioactive contents. Figure 5a,b show the enumeration of *S. boulardii* and *L. plantarum* in fermented oat drink treatments after 24 h of fermentation. The enumeration of probiotics showed an increasing trend in all treatments after 24 h of fermentation. The results indicated that the enzyme treatment supported significantly (*p* < 0.05) better *S. boulardii* cell growth (7.04 Log CFU mL^−1^), compared to the other oat drink fermented with *S. boulardii* treatments. Furthermore, the findings indicated that *L. plantarum* subjected to acidic–enzyme treatment exhibited the highest population density compared to the other oat drink fermented with *L. plantarum* and sequentially fermented oat drink treatments, achieving concentrations of 12.36 and 12.20 Log CFU mL^−1^, respectively.

Nowadays, the use of *S. boulardii* for developing functional plant‐based food is increasing. This strain was employed for the fermentation of rice bran or vegetable juice with an initial concentration ranging from 5.8 to 9 Log CFU mL^−1^ (Değirmencioğlu et al. 2016; Ryan et al. 2011). The results of this study are in agreement with the result of Rekha and Vijayalakshmi (2010), who reported that the population of *S. boulardii* increased (> 7 Log CFU mL^−1^) during the fermentation of soymilk with probiotic bacteria. Additionally, *S. boulardii* was used for SSF of wheat and oat bran by Călinoiu et al. (2019). They reported that the enumeration of the yeast increased to 8.58 Log CFU g^−1^after 4 days of fermentation.

Due to the high genetic and phenotypic diversity of *L. plantarum* , the production of diverse metabolites, higher survivability at low pH, and higher concentration of lactate, this strain was used for the fermentation of different plant‐based foods (Duar et al. 2017; Plengvidhya et al. 2007). *L. plantarum* 299v was first used for producing “Proviva,” the first plant‐based probiotic beverage, with a final concentration of 7 Log CFU mL^−1^ (Prado et al. 2008). GoodBelly (Nextfoods, UK) is another plant‐based beverage that contained 10.3 Log CFU per serving (Gupta and Abu‐Ghannam 2012). The results of this study were higher than the results of Charalampopoulos and Pandiella (2010), Rathore et al. (2012), and also Salmerón et al. (2015), who reported that the enumeration of *L. plantarum* ranged from 7.76 to 10 Log CFU mL^−1^ after fermentation of cereal‐based extract. Also, the enumeration of probiotic bacteria in this study was higher than that reported by Russo et al. (2016), who found that the final concentration of *L. plantarum* , with an initial inoculation of 8 Log CFU mL^−1^, was approximately 9 Log CFU mL^−1^ after 16 h of fermentation of whole oat‐based food.

The results are consistent with those of Gupta et al. (2010), who reported that the enumeration of *L. plantarum* in an oat‐based drink containing sugar reached 10.56 Log CFU mL^−1^ after 8 h of fermentation. However, Gupta and Bajaj (2017) reported a higher enumeration of *L. plantarum* M‐13 after 72 h of fermentation of oat flour (16.9 Log CFU mL^−1^) compared to these results, which may be attributed to the addition of honey. The enumeration of probiotics in different studies may vary due to the genetic variation of probiotics (Rekha and Vijayalakshmi 2010), the composition and condition of growth media such as sugar content, FAN, accumulation of metabolites like lactic acid, and pH value during fermentation (Rozada‐Sánchez et al. 2008), the time and temperature of fermentation, and the physicochemical properties of the food matrix influenced by different pretreatments. It has been reported that all pretreatments that increase the bioavailability of cereal complex nutrients can support the growth of probiotics in media. Although the concentration of sugar and FAN are important factors in microbial fermentation, a higher concentration of FAN (> 220 mg FAN L^−1^) has little effect on microbial growth (Kedia et al. 2007). Due to the high initial concentration of FAN in this study (> 2774 mg FAN L^−1^), the pretreatments that increased sugar concentration had a major effect on microbial growth.

Previous studies have reported the effect of yeast fermentation on the bacterial population during co‐fermentation, but to our knowledge, this is the first study to report the effect of pre‐culturing and inactivating *S. boulardii* on probiotic bacteria during food fermentation. It has been reported that yeast enhances probiotic bacterial growth during mixed fermentation of malt suspension with *Lactobacillus reuteri* (Kedia et al. 2007). Similarly, previous studies reported that co‐fermentation of coffee, beet molasses, and green tea with *S. boulardii* and probiotic *Lactobacillus* enhanced the bacterial growth (Chan et al. 2021; González‐Figueredo et al. 2021; Wang et al. 2022). All of these studies have predominantly focused on the simultaneous fermentation of yeast and bacteria. In this research, the objective was to develop a paraprobiotic–probiotic food product, necessitating yeast fermentation and inactivation prior to probiotic bacterial cultivation. Based on the results of Yang et al. (2025), it was anticipated that yeast fermentation, due to its consumption of nutrient resources, would lead to a reduction in bacterial population growth during the second fermentation phase. However, the findings revealed no significant difference in bacterial counts between the sequentially fermented oat drink and the oat drink fermented with *L. plantarum* . These results indicate that yeast prefermentation supports the proliferation of probiotic bacteria in the subsequent fermentation stage while heat‐inactivated form of *S. boulardii* provided paraprobiotic benefits. The mechanism of supporting the growth of probiotic bacteria by yeast fermentation may be related to the production of nutritional metabolites such as vitamins, FAN, especially amino acids, and other essential nutrients for the growth of *L. plantarum* , which are found in yeast extracts (Kedia et al. 2007; Ponomarova et al. 2017; Rozada‐Sánchez et al. 2008), the disruption of food matrix macromolecules and increasing bioavailability of those due to the exoenzymes limitation of *L. plantarum* (Patel et al. 2008), the increase of buffering properties of growth media (Pawlos et al. 2024), and alleviation of oxidative stress imposed on *L. plantarum* (Odamaki et al. 2011). The acidic–enzyme treatment, which showed a significantly (*p* < 0.05) higher population of *L. plantarum* in oat drink fermented with *L. plantarum* and sequentially fermented oat drink treatments compared to the other treatments, may be due to the growth‐supporting effect of remaining phosphoric acid (Nair et al. 2015), high pH value despite higher acidity due to the buffering properties of metabolites of inactivated yeast (Pawlos et al. 2024) and phosphate salts (Mennah‐Govela and Bornhorst 2021), high initial concentration of the reducing sugar, and the growth‐supporting properties of inactivated yeast. According to the lowest FAN content of the acidic–enzyme treatment of oat drink fermented with *S. boulardii*, which was used for fermentation of *L. plantarum* , the FAN had negligible effects on supporting the growth of probiotic bacteria. The main effects of supporting the growth of inactivated‐fermented yeast on probiotic bacteria may be due to the control of lactic acid production, which led to controlling the pH value along with other factors mentioned above. After 24 h of fermentation, the probiotic bacterial counts of oat drink fermented with *L. plantarum* and sequentially fermented oat drink treatments showed values above 10 Log CFU mL^−1^. According to Palanivelu et al. (2022), probiotic foods should have at least 6–7 Log CFU per mL or g of viable probiotics for better functionality. The results of this study showed that oat drink is a suitable vehicle for developing functional food.

### 3.5 Ethanol Production

Ethanol is an antimicrobial agent produced by *S. boulardii* as the main yeast metabolite. Lactic acid bacteria may be sensitive to ethanol presence depending on the strain, temperature, pH, etc. (Krieger‐Weber et al. 2020). The ethanol concentration of different treatments is presented in Table 1 and ranged from 0.034% (acidic treatment) to 0.302% (sprouting–enzyme treatment) after *S. boulardii* fermentation. Kedia et al. (2007) reported that ethanol (0.73%) was produced by yeast during malt production, which is in agreement with the present findings. Additionally, Senkarcinova et al. (2019) produced beer by using *S. boulardii* fermentation and reported that ethanol was lower than 0.5%, considering it an alcohol‐free beer. The higher ethanol concentration of the sprouting–enzyme treatment may be due to the higher FAN and reducing sugar content, which aligns with Beigbeder et al. (2021) and Thanapornsin et al. (2025), who reported that higher reducing sugar and especially FAN could increase ethanol production yield. The lower ethanol content of the acidic treatment may be due to the production of yeast‐unfermentable sugars by phosphoric acid treatment (Marđetko 2019). The results of this study demonstrated that oat drink fermented with *S. boulardii*, due to the low ethanol production (lower than 0.5%), is considered as a non‐alcoholic beverage (Senkarcinova et al. 2019).

**TABLE 1:** Ethanol concentration of different treatments of oat drinks after *S. boulardii* fermentation (mean ± SD).

Treatments	Ethanol concentration (%)
C	0.133 ± 0.013^c^
Ac	0.034 ± 0.008^a^
Al	0.064 ± 0.010^ab^
En	0.158 ± 0.030^c^
Sp	0.230 ± 0.017^d^
Sp‐Ac	0.242 ± 0.019^d^
Sp‐En	0.302 ± 0.012^e^
En‐Al	0.073 ± 0.010^b^
Ac‐En	0.163 ± 0.0360^c^

*Note:* Treatments: control (C), phosphoric acid (Ac), alkaline (Al), α‐amylase enzyme (En), sprouting (Sp), sprouting‐phosphoric acid (Sp‐Ac), sprouting‐α‐amylase enzyme (Sp‐En), α‐amylase enzyme‐alkaline (En‐Al), and phosphoric acid‐ α‐amylase enzyme (Ac‐En). Values followed by the different letters are significant at the 5% level.

### 3.6 Sensory Evaluation

**FIGURE 6:**
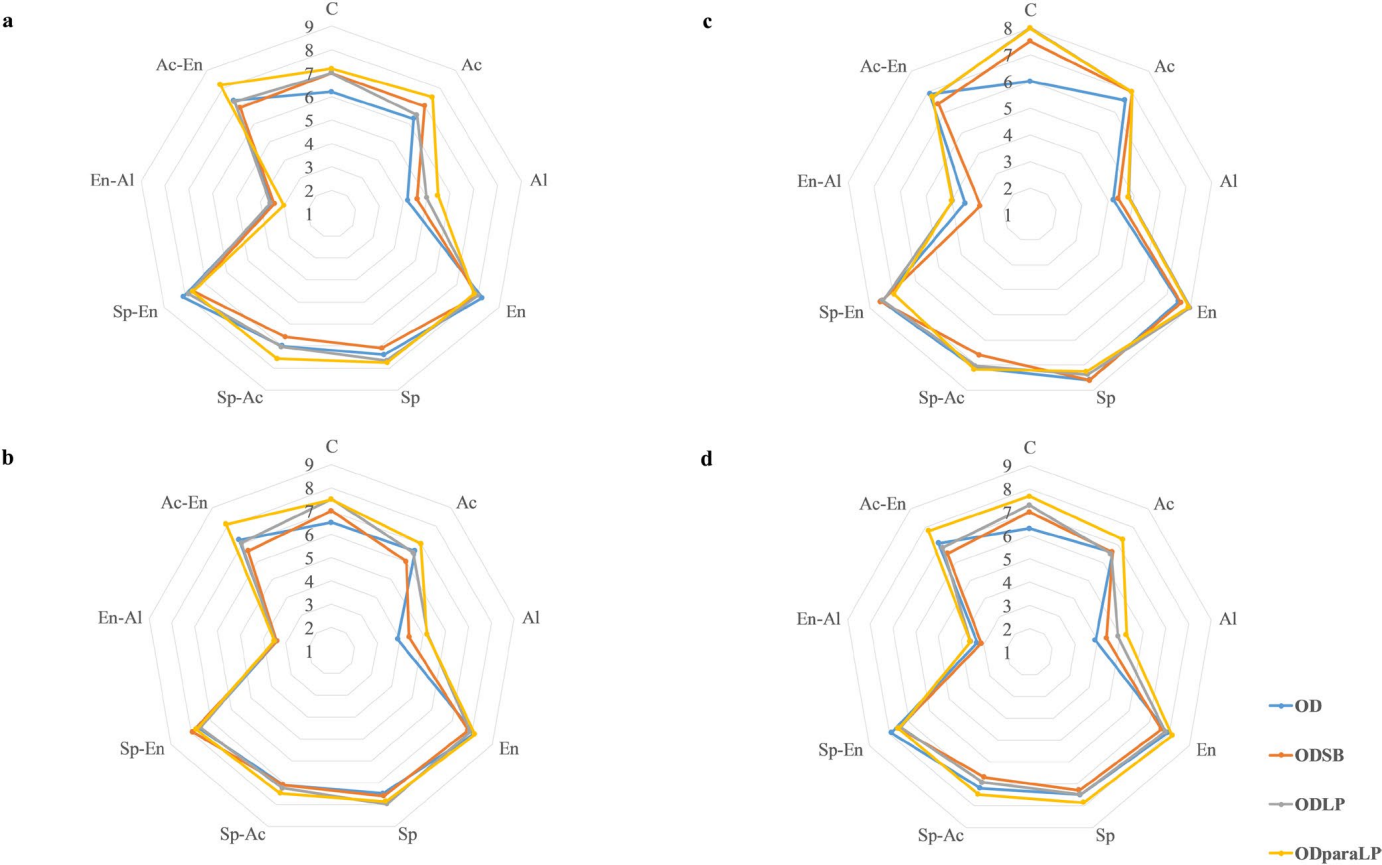
The color (a–d) scores of non‐fermented oat drink (OD), oat drink fermented with *S. boulardii* (ODSB), oat drink fermented with *L. plantarum* (ODLP), and sequential fermented oat drink with *S. boulardii* and *L. plantarum* (ODparaLP); Treatments: Control (C), phosphoric acid (Ac), alkaline (Al), α‐amylase enzyme (En), sprouting (Sp), sprouting‐phosphoric acid (Sp‐Ac), sprouting‐α‐amylase enzyme (Sp‐En), α‐amylase enzyme‐alkaline (En‐Al), and phosphoric acid‐α‐amylase enzyme (Ac‐En).

Besides the chemical factors of a beverage, the sensory evaluation of fermented products is one of the most important factors influencing consumer choices. The results of the sensorial properties such as color, aroma, flavor, and overall acceptability of fermented oat drink treatments are shown in Figure 6a–d, respectively. The mean sensory scores for color, aroma, flavor, and overall acceptability of most fermented oat drinks were higher than 7, which is important for the marketability of the products (Mridula and Sharma 2015). However, in the case of alkaline and enzyme–alkaline treatments, all sensory factors were lower than 6, which is not desirable for marketability. Results showed only slight changes for all sensory characteristics during fermentation methods, but all sensory factors of the control treatment increased after sequentially fermented oat drink treatment and were higher than 7. Additionally, the color, aroma, and overall acceptability of the acidic–enzyme treatment increased after two‐stage fermentation (> 7). It has been reported that the fermentation of oat beverage with *L. plantarum* LP09 enhanced sensory evaluation scores and decreased artificial and earthy tastes (Luana et al. 2014), which aligns with the present findings. These outcomes could be affected by strains of microorganisms (Salmerón et al. 2014), types of pretreatments, acidity, and pH value of the substrate. The use of NaOH for the preparation of alkaline and enzyme–alkaline treatments resulted in the production of a dark oat drink, possibly due to the reaction of phenolic compounds with other oat drink components (Wanasundara et al. 2017). During fermentation, the color of all treatments, especially the alkaline treatment, became brighter due to acid production and decreasing pH value. The enzyme–alkaline treatment showed a darker color even after pH adjustment or fermentation, possibly due to irreversible reactions of phenolic compounds. The higher flavor score of the control treatment may be due to the lower FAN content. The FAN content of oat drink could affect its sensorial properties, induced by free amino acids or peptides (Salmerón et al. 2015). The results showed higher sensorial scores for most sequentially fermented oat drink treatments compared to oat drink fermented with *L. plantarum* treatments, which may be due to the interaction of two microorganisms in sequential fermentation, which produced different metabolites (Kedia et al. 2007), lower acidity, higher pH value, and lower FAN content of those treatments.

## 4 Conclusion

In this study, we developed a functional oat drink with probiotic and paraprobiotic‐probiotic properties using different pretreatments and fermentation methods. We used *S. boulardii* and *L. plantarum* 299v as the probiotic microorganisms and evaluated the effects of fermentation on the nutritional, functional, and sensory characteristics of oat drink. Results indicated that the investigated factors were influenced by the type of fermentation. We found that the pretreatments with α‐amylase and phosphoric acid enhanced the enumeration of *S. boulardii*. Moreover, results showed that phosphoric acid‐α‐amylase and sprouting‐α‐amylase treatments enhanced the *L. plantarum* 299v count in sequential and single fermentation of oat drink. We also found that all types of fermentation increased the phenolic content, antioxidant activity, and sensory scores of oat drink. Moreover, we found that the two‐stage sequential fermentation of oat drink with *S. boulardii* supported the growth of *L. plantarum* 299v and produced a paraprobiotic‐probiotic food with higher sensory evaluation. These results suggest that the pretreatments and fermentation methods used in this study can be applied to produce novel functional foods with improved functional properties and consumer acceptance.

## Author Contributions

**Hassan Gandomi:** conceptualization, methodology, validation, writing – original draft, software, supervision, data curation, funding acquisition, project administration. **Afshin Akhondzadeh Basti:** conceptualization, methodology, data curation, supervision, software, funding acquisition. **Melika Farzaneh:** conceptualization, investigation, methodology. **Sara Ahadzadeh:** conceptualization, investigation, methodology, software, data curation, writing – original draft. **Nima Babolanimogadam:** conceptualization, investigation, funding acquisition, writing – original draft, methodology, validation, software, formal analysis. **Mohammad J. Taherzadeh:** methodology, supervision, conceptualization, data curation, visualization. **Seyed Hasan Sajjadi Alhashem:** conceptualization, investigation, methodology.

## Ethics Statement

All panelists enrolled in this study provided written informed consent. Also, they were informed that they can withdraw from the evaluation at any time without giving a reason.

## Conflicts of Interest

The authors declare no conflicts of interest.

## Data Availability

The data that support the findings of this study are available from the corresponding author upon reasonable request.
